# 2-Anilino-5,7-dimethyl­pyrazolo­[1,5-*a*]pyrimidine-3-carbonitrile

**DOI:** 10.1107/S1600536812035878

**Published:** 2012-08-25

**Authors:** Shaaban K. Mohamed, Mehmet Akkurt, Frank R. Fronczek, Mahmoud A. A. El-Remaily, Antar A. Abdelhamid

**Affiliations:** a Chemistry and Environmental Division, Manchester Metropolitan University, Manchester M1 5GD, England; b Department of Physics, Faculty of Sciences, Erciyes University, 38039 Kayseri, Turkey; c Department of Chemistry, Louisiana State University, Baton Rouge, LA 70803-1804, USA; d Department of Chemistry, Faculty of Science, Sohag University, 82524 Sohag, Egypt

## Abstract

The title compound, C_15_H_13_N_5_, crystallizes with two independent mol­ecules in the asymmetric unit. The mol­ecular conformations are stabilized by C—H⋯N contacts forming *S*(6) ring motifs. In the crystal, pairs of mol­ecules are connected into *R*_2_^2^(12) dimers by N—H⋯N hydrogen bonds. C—H⋯π inter­actions and π–π stacking inter­actions [centroid–centroid distances = 3.6085 (8), 3.6657 (8), 3.4745 (8) and 3.5059 (8) Å] also also observed.

## Related literature

For background details and bioactivity of pyrazolo­pyrimidine compounds, see: Singh *et al.* (2011 ▶); Rajendran *et al.* (2011 ▶); Earl *et al.*(1975 ▶); Bendich *et al.* (1954 ▶); Elion *et al.* (1963 ▶); Hildick & Shaw (1971 ▶); Kabayasahi (1973 ▶); Satherland *et al.* (1968 ▶); Soliman *et al.* (2010 ▶). For bond-length data, see: Allen *et al.* (1987 ▶). For hydrogen-bond motifs, see: Bernstein *et al.* (1995 ▶).
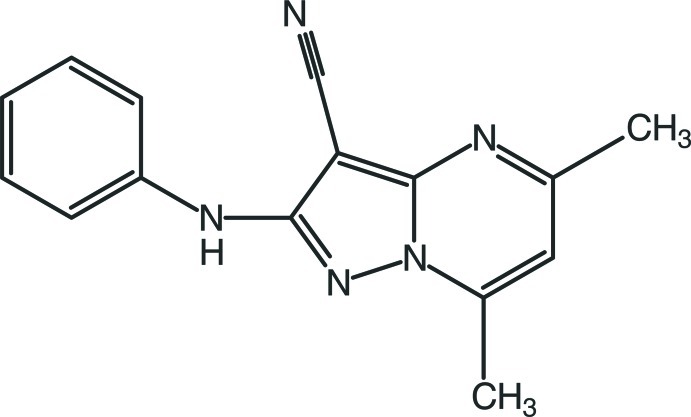


## Experimental

### 

#### Crystal data

C_15_H_13_N_5_
*M* *_r_* = 263.30
Monoclinic, 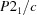

*a* = 11.2139 (4) Å
*b* = 10.4347 (4) Å
*c* = 22.1753 (9) Å
β = 94.569 (1)°
*V* = 2586.57 (17) Å^3^
*Z* = 8
Mo *K*α radiation
μ = 0.09 mm^−1^
*T* = 90 K
0.30 × 0.14 × 0.13 mm

#### Data collection

Bruker Kappa APEXII DUO diffractometer
Absorption correction: multi-scan (*SADABS*; Sheldrick, 2004 ▶) *T*_min_ = 0.975, *T*_max_ = 0.989
16931 measured reflections
6402 independent reflections
4813 reflections with *I* > 2σ(*I*)
*R*_int_ = 0.027
Standard reflections: 0

#### Refinement

*R*[*F* ^2^ > 2σ(*F* ^2^)] = 0.042
*wR*(*F* ^2^) = 0.116
*S* = 1.02
6402 reflections
365 parameters
H-atom parameters constrained
Δρ_max_ = 0.30 e Å^−3^
Δρ_min_ = −0.21 e Å^−3^

### 

Data collection: *APEX2* (Bruker, 2007 ▶); cell refinement: *SAINT* (Bruker, 2007 ▶); data reduction: *SAINT*; program(s) used to solve structure: *SIR97* (Altomare *et al.*, 1999 ▶); program(s) used to refine structure: *SHELXL97* (Sheldrick, 2008 ▶); molecular graphics: *ORTEP-3 for Windows* (Farrugia, 1997 ▶) and *PLATON* (Spek, 2009 ▶); software used to prepare material for publication: *WinGX* (Farrugia, 1999 ▶) and *PLATON*.

## Supplementary Material

Crystal structure: contains datablock(s) global, I. DOI: 10.1107/S1600536812035878/bt6821sup1.cif

Structure factors: contains datablock(s) I. DOI: 10.1107/S1600536812035878/bt6821Isup2.hkl

Supplementary material file. DOI: 10.1107/S1600536812035878/bt6821Isup3.cml

Additional supplementary materials:  crystallographic information; 3D view; checkCIF report

## Figures and Tables

**Table 1:** Hydrogen-bond geometry (Å, °) *Cg*1 and *Cg*3 are the centroids of the N2/N3/C7–C9 and N3/N5/C9/C11–C13 rings, respectively.

*D*—H⋯*A*	*D*—H	H⋯*A*	*D*⋯*A*	*D*—H⋯*A*
N1—H1*A*⋯N9^i^	0.86	2.20	3.0487 (17)	168
N6—H6⋯N4^ii^	0.86	2.23	3.0703 (17)	167
C1—H1⋯N2	0.93	2.40	2.9158 (17)	115
C16—H16⋯N7	0.93	2.28	2.9059 (17)	124
C4—H4⋯*Cg*1^iii^	0.93	2.83	3.5730 (16)	138
C27—H27⋯*Cg*3^iii^	0.93	2.99	3.7462 (15)	139
C29—H29*B*⋯*Cg*3	0.96	2.95	3.7420 (16)	141

Symmetry codes: (i) 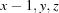; (ii) 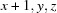; (iii) 
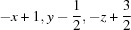
.

**Fig. 1.**
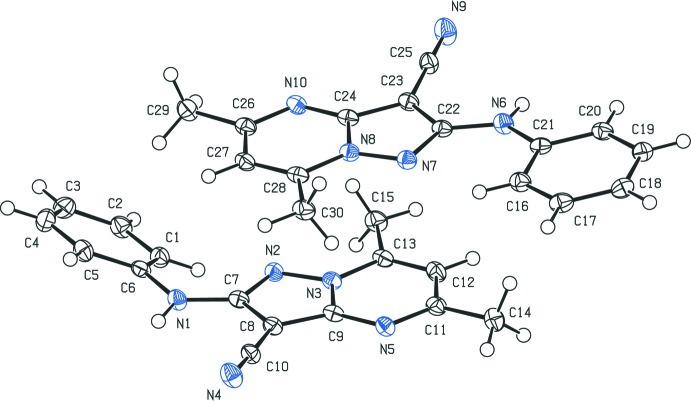
View of two independent molecules of C15H13N5 in the asymmetric unit. Displacement ellipsoids for non-H atoms are drawn at the 50% probability level.

**Fig. 2.**
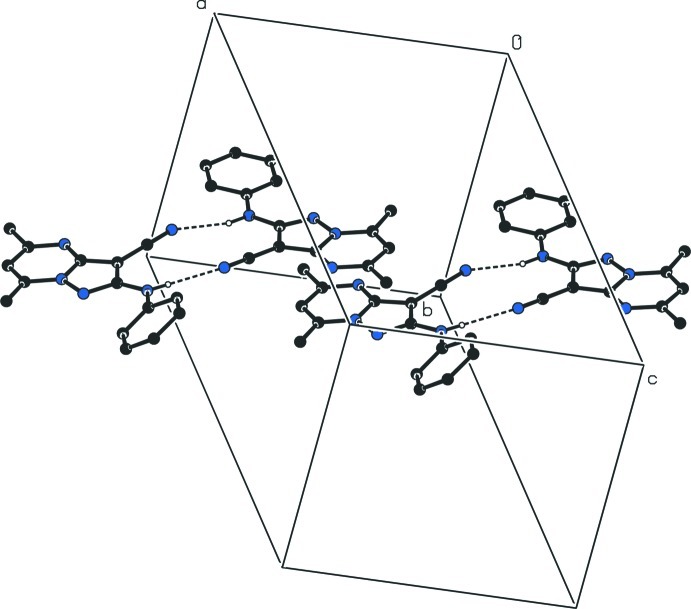
View of the dimers formed by pairs of N—H···N hydrogen bonds, forming a R22(12) motif. H atoms not involved in hydrogen bonds have been omitted for clarity.

## supplementary crystallographic information

### Comment

The pyrazole containing compounds have practical applications in the medicinal and agrochemical fields. Pyrazolopyrimidines in particular represent a class of nitrogen bridgehead antibiotic compounds such as formycin and and allopurinol (Elion *et al.*, 1963) which are still the drug of choice for the treatment of hyperurecemia and gouty arthritis (Earl *et al.*, 1975). In addition, pyrazolopyrimidines have exhibited antimicrobial (Singh *et al.*, 2011; Rajendran *et al.* 2011, Hildick & Shaw, 1971), antitumor and antileukemic activities (Earl *et al.*, 1975). They are as purine analogues (Bendich *et al.*, 1954; Kabayasahi, 1973) and as such they have useful properties as antimetabolites in purine biochemical reaction (Satherland *et al.*, 1968). In view of the importance of pyrazolpyrimidine derivatives we have planned a systematic study of such compounds, and describe here the crystal structure of the title compound (I) as one of potential bioactive derivative.

As shown in Fig. 1, the two independent molecules (A with N1 and B with N6) in the asymmetric unit of (I) have a similar conformation. In both molecules (A and B), the pyrazolo[1,5-*a*]pyrimidine rings are essentially planar [maximum deviaitions are 0.015 (1) Å for N2 and N3 in molecule A, and 0.018 (1) Å for N7 in molecule B]. The dihedral angles between the pyrazolo[1,5-*a*]pyrimidine and phenyl rings are 29.74 (6)° in molecule A and 3.34 (6)° in molecule B. In the both molecules (A and B) of (I), the bond lengths and bond angles are found to have normal values (Allen *et al.*, 1987).

Molecular conformations of the molecules A and B are stabilized by C—H···N contacts generating S(6) ring motifs (Table 1). In the crystal, *R*_2_^2^(12) dimers by N—H···N hydrogen bonds connect pairs of molecules to each other (Bernstein *et al.*,1995; Table 1, Fig. 2). Furthermore, C—H···π interactions and π-π stacking interactions [*Cg*1···*Cg*6 (*x*, *y*, *z*) = 3.6085 (8) Å, *Cg*2···*Cg*5 (*x*, *y*, *z*) = 3.6657 (8) Å, *Cg*5···*Cg*7 (2 - *x*, -*y*, 1 - *z*) = 3.4745 (8) Å and *Cg*6···*Cg*7 (2 - *x*, -*y*, 1 - *z*) = 3.5059 (8) Å; where *Cg*1, *Cg*2, *Cg*5, *Cg*6 and *Cg*7 are the N2/N3/C7–C9, N3/N5/C9/C11–C13, N7/N8/C22–C24, N8/N10/C24/C26–C28 and C16–C21 rings, respectively] are also observed in the crystal structure.

### Experimental

The title compound was prepared according to the reported literature (Soliman *et al.*, 2010). Suitable crystals for X-ray diffraction were obtained from ethanol solution of (I) by slow evaporation method (*M*.p.: 553 K).

### Refinement

All H atoms were positioned geometrically and refined using a riding model with N—H = 0.86 Å, C—H = 0.93 and 0.96 Å, and with *U*_iso_ = 1.2 or 1.5*U*_eq_(C,*N*).

### Crystal data

**Table:**

C_15_H_13_N_5_	*F*(000) = 1104
*M**_r_* = 263.30	*D*_x_ = 1.352 Mg m^−^^3^
Monoclinic, *P*2_1_/*c*	Mo *K*α radiation, λ = 0.71073 Å
Hall symbol: -P 2ybc	Cell parameters from 5253 reflections
*a* = 11.2139 (4) Å	θ = 2.5–28.3°
*b* = 10.4347 (4) Å	µ = 0.09 mm^−^^1^
*c* = 22.1753 (9) Å	*T* = 90 K
β = 94.569 (1)°	Needle, yellow
*V* = 2586.57 (17) Å^3^	0.30 × 0.14 × 0.13 mm
*Z* = 8	

### Data collection

**Table:**

Bruker Kappa APEXII DUO diffractometer	6402 independent reflections
Radiation source: sealed tube	4813 reflections with *I* > 2σ(*I*)
TRIUMPH curved graphite monochromator	*R*_int_ = 0.027
φ and ω scans	θ_max_ = 28.3°, θ_min_ = 1.8°
Absorption correction: multi-scan (*SADABS*; Sheldrick, 2004)	*h* = −14→14
*T*_min_ = 0.975, *T*_max_ = 0.989	*k* = −9→13
16931 measured reflections	*l* = −29→29

### Refinement

**Table:**

Refinement on *F*^2^	Primary atom site location: structure-invariant direct methods
Least-squares matrix: full	Secondary atom site location: difference Fourier map
*R*[*F*^2^ > 2σ(*F*^2^)] = 0.042	Hydrogen site location: inferred from neighbouring sites
*wR*(*F*^2^) = 0.116	H-atom parameters constrained
*S* = 1.02	*w* = 1/[σ^2^(*F*_o_^2^) + (0.0561*P*)^2^ + 0.7958*P*] where *P* = (*F*_o_^2^ + 2*F*_c_^2^)/3
6402 reflections	(Δ/σ)_max_ = 0.001
365 parameters	Δρ_max_ = 0.30 e Å^−^^3^
0 restraints	Δρ_min_ = −0.21 e Å^−^^3^

### Special details

**Table:**

Geometry. Bond distances, angles *etc*. have been calculated using the rounded fractional coordinates. All su's are estimated from the variances of the (full) variance-covariance matrix. The cell e.s.d.'s are taken into account in the estimation of distances, angles and torsion angles
Refinement. Refinement on *F*^2^ for ALL reflections except those flagged by the user for potential systematic errors. Weighted *R*-factors *wR* and all goodnesses of fit *S* are based on *F*^2^, conventional *R*-factors *R* are based on *F*, with *F* set to zero for negative *F*^2^. The observed criterion of *F*^2^ > σ(*F*^2^) is used only for calculating -*R*-factor-obs *etc*. and is not relevant to the choice of reflections for refinement. *R*-factors based on *F*^2^ are statistically about twice as large as those based on *F*, and *R*-factors based on ALL data will be even larger.

### Fractional atomic coordinates and isotropic or equivalent isotropic displacement parameters (Å^2^)

**Table:**

	*x*	*y*	*z*	*U*_iso_*/*U*_eq_	
N1	0.47831 (10)	0.32101 (12)	0.70318 (5)	0.0199 (3)	
N2	0.66448 (10)	0.40025 (11)	0.67523 (5)	0.0180 (3)	
N3	0.70882 (10)	0.41926 (11)	0.61962 (5)	0.0166 (3)	
N4	0.33625 (11)	0.25331 (13)	0.54197 (6)	0.0266 (4)	
N5	0.65371 (10)	0.40210 (11)	0.51345 (5)	0.0192 (3)	
C1	0.58840 (12)	0.39461 (14)	0.79791 (6)	0.0206 (4)	
C2	0.60952 (12)	0.38176 (15)	0.86009 (6)	0.0231 (4)	
C3	0.55016 (13)	0.28965 (15)	0.89136 (7)	0.0255 (4)	
C4	0.46676 (13)	0.21140 (14)	0.85998 (7)	0.0251 (4)	
C5	0.44404 (12)	0.22347 (14)	0.79814 (6)	0.0211 (4)	
C6	0.50542 (11)	0.31412 (13)	0.76635 (6)	0.0178 (4)	
C7	0.55347 (11)	0.35638 (13)	0.66085 (6)	0.0174 (4)	
C8	0.52493 (11)	0.34861 (13)	0.59719 (6)	0.0178 (4)	
C9	0.62799 (11)	0.39049 (13)	0.57116 (6)	0.0173 (4)	
C10	0.42096 (12)	0.29756 (13)	0.56611 (6)	0.0193 (4)	
C11	0.76337 (12)	0.44325 (13)	0.50442 (6)	0.0198 (4)	
C12	0.84888 (12)	0.47222 (13)	0.55295 (6)	0.0205 (4)	
C13	0.82237 (12)	0.45941 (13)	0.61182 (6)	0.0186 (4)	
C14	0.79399 (13)	0.45550 (15)	0.44007 (7)	0.0246 (4)	
C15	0.90416 (12)	0.48200 (14)	0.66696 (6)	0.0226 (4)	
N6	1.07117 (10)	0.19315 (11)	0.50864 (5)	0.0198 (3)	
N7	0.87866 (10)	0.13453 (11)	0.53749 (5)	0.0184 (3)	
N8	0.83415 (10)	0.11918 (11)	0.59317 (5)	0.0171 (3)	
N9	1.22465 (11)	0.22274 (14)	0.67265 (6)	0.0304 (4)	
N10	0.89182 (10)	0.13317 (11)	0.69927 (5)	0.0186 (3)	
C16	0.94456 (12)	0.15164 (13)	0.41364 (6)	0.0210 (4)	
C17	0.93656 (13)	0.14738 (14)	0.35064 (7)	0.0229 (4)	
C18	1.03505 (13)	0.17251 (14)	0.31852 (7)	0.0227 (4)	
C19	1.14369 (13)	0.20196 (13)	0.35019 (6)	0.0216 (4)	
C20	1.15306 (12)	0.20798 (13)	0.41256 (6)	0.0193 (4)	
C21	1.05356 (12)	0.18282 (12)	0.44522 (6)	0.0181 (4)	
C22	0.99284 (12)	0.16850 (12)	0.55138 (6)	0.0173 (4)	
C23	1.02218 (12)	0.17663 (13)	0.61492 (6)	0.0180 (4)	
C24	0.91709 (11)	0.14323 (13)	0.64140 (6)	0.0173 (4)	
C25	1.13379 (12)	0.20222 (14)	0.64679 (6)	0.0206 (4)	
C26	0.78134 (12)	0.09541 (13)	0.70864 (6)	0.0194 (4)	
C27	0.69422 (12)	0.06867 (13)	0.66062 (6)	0.0193 (4)	
C28	0.72062 (12)	0.08007 (13)	0.60174 (6)	0.0188 (4)	
C29	0.75352 (13)	0.07701 (15)	0.77310 (7)	0.0240 (4)	
C30	0.63923 (12)	0.05188 (14)	0.54696 (7)	0.0224 (4)	
H1	0.62910	0.45630	0.77730	0.0250*	
H1A	0.40660	0.30070	0.69000	0.0240*	
H2	0.66430	0.43570	0.88110	0.0280*	
H3	0.56610	0.28050	0.93290	0.0310*	
H4	0.42580	0.15020	0.88080	0.0300*	
H5	0.38750	0.17090	0.77760	0.0250*	
H12	0.92450	0.50050	0.54470	0.0250*	
H14A	0.84830	0.38830	0.43100	0.0370*	
H14B	0.83090	0.53720	0.43440	0.0370*	
H14C	0.72230	0.44880	0.41350	0.0370*	
H15A	0.86990	0.54510	0.69190	0.0340*	
H15B	0.98000	0.51210	0.65540	0.0340*	
H15C	0.91540	0.40340	0.68920	0.0340*	
H6	1.14110	0.21850	0.52240	0.0240*	
H16	0.87780	0.13390	0.43450	0.0250*	
H17	0.86380	0.12730	0.32970	0.0280*	
H18	1.02860	0.16970	0.27650	0.0270*	
H19	1.21060	0.21770	0.32910	0.0260*	
H20	1.22600	0.22890	0.43310	0.0230*	
H27	0.61800	0.04300	0.66920	0.0230*	
H29A	0.76610	−0.01110	0.78440	0.0360*	
H29B	0.67160	0.09970	0.77730	0.0360*	
H29C	0.80500	0.13060	0.79890	0.0360*	
H30A	0.56110	0.03090	0.55890	0.0340*	
H30B	0.67020	−0.01920	0.52560	0.0340*	
H30C	0.63390	0.12580	0.52110	0.0340*	

### Atomic displacement parameters (Å^2^)

**Table:**

	*U*^11^	*U*^22^	*U*^33^	*U*^12^	*U*^13^	*U*^23^
N1	0.0128 (5)	0.0291 (7)	0.0172 (6)	−0.0027 (5)	−0.0017 (4)	−0.0004 (5)
N2	0.0161 (5)	0.0196 (6)	0.0183 (6)	−0.0002 (4)	0.0009 (4)	0.0004 (4)
N3	0.0149 (5)	0.0172 (6)	0.0174 (6)	0.0003 (4)	−0.0004 (4)	−0.0020 (4)
N4	0.0188 (6)	0.0376 (8)	0.0231 (6)	−0.0026 (5)	0.0001 (5)	−0.0049 (5)
N5	0.0175 (5)	0.0210 (6)	0.0192 (6)	0.0012 (5)	0.0025 (4)	−0.0021 (4)
C1	0.0170 (6)	0.0245 (7)	0.0201 (7)	−0.0016 (5)	0.0010 (5)	0.0012 (5)
C2	0.0181 (6)	0.0303 (8)	0.0205 (7)	−0.0003 (6)	−0.0015 (5)	−0.0026 (6)
C3	0.0265 (7)	0.0327 (8)	0.0169 (7)	0.0040 (6)	−0.0006 (5)	0.0022 (6)
C4	0.0283 (7)	0.0240 (7)	0.0238 (7)	−0.0004 (6)	0.0068 (6)	0.0036 (6)
C5	0.0188 (6)	0.0215 (7)	0.0233 (7)	−0.0020 (5)	0.0032 (5)	−0.0033 (5)
C6	0.0137 (6)	0.0216 (7)	0.0180 (6)	0.0042 (5)	0.0003 (5)	−0.0007 (5)
C7	0.0152 (6)	0.0170 (6)	0.0196 (7)	0.0012 (5)	−0.0011 (5)	−0.0018 (5)
C8	0.0146 (6)	0.0200 (7)	0.0185 (7)	0.0009 (5)	−0.0003 (5)	−0.0016 (5)
C9	0.0144 (6)	0.0167 (6)	0.0203 (7)	0.0022 (5)	−0.0015 (5)	−0.0028 (5)
C10	0.0166 (6)	0.0237 (7)	0.0177 (7)	0.0017 (5)	0.0021 (5)	−0.0019 (5)
C11	0.0197 (7)	0.0176 (7)	0.0222 (7)	0.0027 (5)	0.0029 (5)	−0.0019 (5)
C12	0.0155 (6)	0.0195 (7)	0.0269 (7)	−0.0014 (5)	0.0034 (5)	−0.0017 (5)
C13	0.0139 (6)	0.0151 (6)	0.0266 (7)	0.0006 (5)	−0.0002 (5)	−0.0019 (5)
C14	0.0233 (7)	0.0269 (8)	0.0242 (7)	0.0004 (6)	0.0064 (6)	−0.0020 (6)
C15	0.0166 (6)	0.0251 (7)	0.0253 (7)	−0.0032 (6)	−0.0037 (5)	−0.0011 (6)
N6	0.0148 (5)	0.0241 (6)	0.0198 (6)	−0.0035 (5)	−0.0022 (4)	−0.0022 (5)
N7	0.0165 (5)	0.0181 (6)	0.0204 (6)	−0.0010 (4)	0.0001 (4)	−0.0005 (4)
N8	0.0147 (5)	0.0171 (6)	0.0191 (6)	−0.0004 (4)	−0.0015 (4)	−0.0010 (4)
N9	0.0208 (6)	0.0462 (8)	0.0237 (7)	−0.0064 (6)	−0.0005 (5)	−0.0009 (6)
N10	0.0158 (5)	0.0194 (6)	0.0205 (6)	0.0005 (4)	0.0002 (4)	−0.0027 (4)
C16	0.0181 (6)	0.0197 (7)	0.0247 (7)	−0.0008 (5)	−0.0008 (5)	0.0000 (5)
C17	0.0214 (7)	0.0221 (7)	0.0240 (7)	−0.0012 (6)	−0.0063 (5)	0.0000 (6)
C18	0.0263 (7)	0.0217 (7)	0.0194 (7)	0.0010 (6)	−0.0020 (5)	0.0013 (5)
C19	0.0207 (7)	0.0201 (7)	0.0240 (7)	0.0014 (5)	0.0016 (5)	0.0026 (5)
C20	0.0162 (6)	0.0182 (7)	0.0228 (7)	0.0008 (5)	−0.0023 (5)	0.0002 (5)
C21	0.0190 (6)	0.0141 (6)	0.0209 (7)	0.0026 (5)	−0.0011 (5)	−0.0001 (5)
C22	0.0162 (6)	0.0143 (6)	0.0209 (7)	0.0009 (5)	−0.0008 (5)	−0.0008 (5)
C23	0.0150 (6)	0.0181 (6)	0.0205 (7)	0.0002 (5)	−0.0011 (5)	−0.0017 (5)
C24	0.0151 (6)	0.0145 (6)	0.0217 (7)	0.0011 (5)	−0.0021 (5)	−0.0024 (5)
C25	0.0175 (6)	0.0248 (7)	0.0195 (7)	−0.0022 (5)	0.0022 (5)	−0.0004 (5)
C26	0.0177 (6)	0.0161 (6)	0.0244 (7)	0.0026 (5)	0.0012 (5)	−0.0015 (5)
C27	0.0134 (6)	0.0177 (7)	0.0267 (7)	0.0004 (5)	0.0011 (5)	−0.0005 (5)
C28	0.0144 (6)	0.0145 (6)	0.0270 (7)	0.0014 (5)	−0.0020 (5)	−0.0010 (5)
C29	0.0194 (7)	0.0290 (8)	0.0237 (7)	0.0008 (6)	0.0026 (5)	−0.0013 (6)
C30	0.0163 (6)	0.0235 (7)	0.0266 (7)	−0.0020 (6)	−0.0036 (5)	−0.0007 (6)

### Geometric parameters (Å, º)

**Table:**

N1—C6	1.4114 (17)	C3—H3	0.9300
N1—C7	1.3615 (17)	C4—H4	0.9300
N2—N3	1.3802 (16)	C5—H5	0.9300
N2—C7	1.3408 (17)	C12—H12	0.9300
N3—C9	1.3825 (17)	C14—H14C	0.9600
N3—C13	1.3645 (18)	C14—H14B	0.9600
N4—C10	1.1494 (19)	C14—H14A	0.9600
N5—C9	1.3396 (17)	C15—H15A	0.9600
N5—C11	1.3326 (18)	C15—H15B	0.9600
N1—H1A	0.8600	C15—H15C	0.9600
N6—C21	1.4086 (17)	C16—C21	1.3978 (19)
N6—C22	1.3670 (17)	C16—C17	1.394 (2)
N7—C22	1.3406 (18)	C17—C18	1.386 (2)
N7—N8	1.3775 (16)	C18—C19	1.391 (2)
N8—C28	1.3646 (18)	C19—C20	1.3801 (19)
N8—C24	1.3833 (17)	C20—C21	1.4026 (19)
N9—C25	1.1488 (19)	C22—C23	1.4237 (19)
N10—C26	1.3320 (18)	C23—C25	1.4129 (19)
N10—C24	1.3402 (17)	C23—C24	1.4019 (18)
N6—H6	0.8600	C26—C27	1.4144 (19)
C1—C2	1.3868 (19)	C26—C29	1.500 (2)
C1—C6	1.3991 (19)	C27—C28	1.3668 (19)
C2—C3	1.386 (2)	C28—C30	1.490 (2)
C3—C4	1.386 (2)	C16—H16	0.9300
C4—C5	1.380 (2)	C17—H17	0.9300
C5—C6	1.3938 (19)	C18—H18	0.9300
C7—C8	1.4247 (19)	C19—H19	0.9300
C8—C10	1.4107 (19)	C20—H20	0.9300
C8—C9	1.4022 (18)	C27—H27	0.9300
C11—C14	1.500 (2)	C29—H29A	0.9600
C11—C12	1.4158 (19)	C29—H29B	0.9600
C12—C13	1.3683 (19)	C29—H29C	0.9600
C13—C15	1.4877 (19)	C30—H30A	0.9600
C1—H1	0.9300	C30—H30B	0.9600
C2—H2	0.9300	C30—H30C	0.9600
			
C6—N1—C7	127.00 (11)	H14B—C14—H14C	109.00
N3—N2—C7	103.32 (10)	C11—C14—H14A	110.00
N2—N3—C9	113.75 (11)	C13—C15—H15A	109.00
N2—N3—C13	124.31 (11)	H15B—C15—H15C	109.00
C9—N3—C13	121.92 (11)	H15A—C15—H15B	109.00
C9—N5—C11	116.37 (11)	H15A—C15—H15C	109.00
C6—N1—H1A	117.00	C13—C15—H15B	109.00
C7—N1—H1A	116.00	C13—C15—H15C	110.00
C21—N6—C22	129.13 (11)	C17—C16—C21	119.58 (13)
N8—N7—C22	103.44 (10)	C16—C17—C18	121.26 (14)
N7—N8—C28	124.63 (11)	C17—C18—C19	118.94 (14)
C24—N8—C28	121.59 (11)	C18—C19—C20	120.64 (13)
N7—N8—C24	113.74 (11)	C19—C20—C21	120.58 (13)
C24—N10—C26	116.28 (11)	N6—C21—C20	116.56 (12)
C22—N6—H6	115.00	N6—C21—C16	124.43 (12)
C21—N6—H6	115.00	C16—C21—C20	119.00 (12)
C2—C1—C6	119.57 (13)	N6—C22—C23	124.36 (12)
C1—C2—C3	120.91 (13)	N6—C22—N7	123.04 (12)
C2—C3—C4	119.27 (14)	N7—C22—C23	112.60 (12)
C3—C4—C5	120.57 (14)	C24—C23—C25	125.32 (12)
C4—C5—C6	120.32 (13)	C22—C23—C25	129.20 (12)
C1—C6—C5	119.34 (12)	C22—C23—C24	105.31 (12)
N1—C6—C1	123.23 (12)	N8—C24—N10	123.07 (11)
N1—C6—C5	117.42 (12)	N8—C24—C23	104.90 (11)
N1—C7—C8	124.41 (12)	N10—C24—C23	132.03 (12)
N2—C7—C8	112.71 (11)	N9—C25—C23	179.83 (16)
N1—C7—N2	122.86 (12)	C27—C26—C29	120.58 (12)
C9—C8—C10	126.36 (12)	N10—C26—C27	122.42 (12)
C7—C8—C9	105.23 (11)	N10—C26—C29	116.96 (12)
C7—C8—C10	128.12 (12)	C26—C27—C28	120.85 (12)
N3—C9—N5	123.04 (11)	C27—C28—C30	126.63 (12)
N5—C9—C8	131.97 (12)	N8—C28—C27	115.76 (12)
N3—C9—C8	104.98 (11)	N8—C28—C30	117.60 (12)
N4—C10—C8	178.10 (15)	C17—C16—H16	120.00
N5—C11—C12	122.13 (12)	C21—C16—H16	120.00
N5—C11—C14	117.03 (12)	C16—C17—H17	119.00
C12—C11—C14	120.83 (12)	C18—C17—H17	119.00
C11—C12—C13	121.26 (12)	C17—C18—H18	121.00
C12—C13—C15	127.02 (12)	C19—C18—H18	121.00
N3—C13—C15	117.70 (12)	C18—C19—H19	120.00
N3—C13—C12	115.26 (12)	C20—C19—H19	120.00
C6—C1—H1	120.00	C19—C20—H20	120.00
C2—C1—H1	120.00	C21—C20—H20	120.00
C3—C2—H2	120.00	C26—C27—H27	120.00
C1—C2—H2	120.00	C28—C27—H27	120.00
C4—C3—H3	120.00	C26—C29—H29A	110.00
C2—C3—H3	120.00	C26—C29—H29B	110.00
C5—C4—H4	120.00	C26—C29—H29C	109.00
C3—C4—H4	120.00	H29A—C29—H29B	109.00
C4—C5—H5	120.00	H29A—C29—H29C	109.00
C6—C5—H5	120.00	H29B—C29—H29C	109.00
C13—C12—H12	119.00	C28—C30—H30A	109.00
C11—C12—H12	119.00	C28—C30—H30B	109.00
H14A—C14—H14B	109.00	C28—C30—H30C	109.00
H14A—C14—H14C	109.00	H30A—C30—H30B	109.00
C11—C14—H14B	110.00	H30A—C30—H30C	109.00
C11—C14—H14C	109.00	H30B—C30—H30C	109.00
			
C7—N1—C6—C1	31.2 (2)	C2—C1—C6—N1	179.79 (13)
C7—N1—C6—C5	−149.89 (14)	C2—C1—C6—C5	0.9 (2)
C6—N1—C7—N2	−5.5 (2)	C6—C1—C2—C3	0.5 (2)
C6—N1—C7—C8	173.05 (13)	C1—C2—C3—C4	−1.4 (2)
C7—N2—N3—C13	−177.38 (12)	C2—C3—C4—C5	0.9 (2)
N3—N2—C7—C8	−0.88 (15)	C3—C4—C5—C6	0.4 (2)
C7—N2—N3—C9	1.00 (15)	C4—C5—C6—N1	179.70 (13)
N3—N2—C7—N1	177.84 (12)	C4—C5—C6—C1	−1.4 (2)
N2—N3—C9—N5	−179.79 (12)	N2—C7—C8—C9	0.48 (16)
N2—N3—C13—C12	179.92 (13)	N1—C7—C8—C10	−4.3 (2)
C9—N3—C13—C12	1.67 (19)	N2—C7—C8—C10	174.45 (13)
C13—N3—C9—N5	−1.4 (2)	N1—C7—C8—C9	−178.22 (13)
C9—N3—C13—C15	−177.18 (12)	C7—C8—C9—N3	0.15 (15)
N2—N3—C9—C8	−0.73 (15)	C10—C8—C9—N3	−173.96 (13)
N2—N3—C13—C15	1.07 (19)	C10—C8—C9—N5	5.0 (2)
C13—N3—C9—C8	177.69 (12)	C7—C8—C9—N5	179.09 (14)
C9—N5—C11—C14	179.40 (12)	C14—C11—C12—C13	−178.97 (13)
C9—N5—C11—C12	0.55 (19)	N5—C11—C12—C13	−0.2 (2)
C11—N5—C9—N3	0.18 (19)	C11—C12—C13—N3	−0.9 (2)
C11—N5—C9—C8	−178.60 (14)	C11—C12—C13—C15	177.78 (13)
C21—N6—C22—C23	176.95 (13)	C21—C16—C17—C18	0.5 (2)
C22—N6—C21—C16	4.5 (2)	C17—C16—C21—C20	−0.6 (2)
C21—N6—C22—N7	−3.5 (2)	C17—C16—C21—N6	178.31 (13)
C22—N6—C21—C20	−176.57 (13)	C16—C17—C18—C19	0.3 (2)
N8—N7—C22—N6	179.77 (12)	C17—C18—C19—C20	−1.0 (2)
C22—N7—N8—C24	0.31 (14)	C18—C19—C20—C21	0.9 (2)
N8—N7—C22—C23	−0.64 (14)	C19—C20—C21—C16	−0.1 (2)
C22—N7—N8—C28	−177.66 (12)	C19—C20—C21—N6	−179.07 (12)
N7—N8—C28—C30	0.07 (19)	N6—C22—C23—C25	−4.3 (2)
N7—N8—C24—C23	0.13 (15)	N7—C22—C23—C25	176.09 (14)
C28—N8—C24—N10	−1.7 (2)	N6—C22—C23—C24	−179.68 (12)
N7—N8—C24—N10	−179.72 (12)	N7—C22—C23—C24	0.74 (15)
C24—N8—C28—C27	1.12 (19)	C22—C23—C24—N8	−0.49 (14)
C28—N8—C24—C23	178.17 (12)	C22—C23—C24—N10	179.33 (14)
C24—N8—C28—C30	−177.75 (12)	C25—C23—C24—N8	−176.07 (13)
N7—N8—C28—C27	178.94 (12)	C25—C23—C24—N10	3.8 (2)
C26—N10—C24—N8	1.42 (19)	C29—C26—C27—C28	−177.22 (13)
C26—N10—C24—C23	−178.38 (14)	N10—C26—C27—C28	0.4 (2)
C24—N10—C26—C29	176.88 (12)	C26—C27—C28—N8	−0.5 (2)
C24—N10—C26—C27	−0.77 (19)	C26—C27—C28—C30	178.25 (13)

### Hydrogen-bond geometry (Å, º)

Cg1 and Cg3 are the centroids of the N2/N3/C7–C9 and N3/N5/C9/C11–C13 rings, respectively.

**Table:**

*D*—H···*A*	*D*—H	H···*A*	*D*···*A*	*D*—H···*A*
N1—H1*A*···N9^i^	0.86	2.20	3.0487 (17)	168
N6—H6···N4^ii^	0.86	2.23	3.0703 (17)	167
C1—H1···N2	0.93	2.40	2.9158 (17)	115
C16—H16···N7	0.93	2.28	2.9059 (17)	124
C4—H4···*Cg*1^iii^	0.93	2.83	3.5730 (16)	138
C27—H27···*Cg*3^iii^	0.93	2.99	3.7462 (15)	139
C29—H29*B*···*Cg*3	0.96	2.95	3.7420 (16)	141

Symmetry codes: (i) *x*−1, *y*, *z*; (ii) *x*+1, *y*, *z*; (iii) −*x*+1, *y*−1/2, −*z*+3/2.
